# Aberrant Mitochondrial Dynamics: An Emerging Pathogenic Driver of Abdominal Aortic Aneurysm

**DOI:** 10.1155/2021/6615400

**Published:** 2021-06-16

**Authors:** Mingqi Ouyang, Mi Wang, Bilian Yu

**Affiliations:** Department of Cardiovascular Medicine, The Second Xiangya Hospital, Central South University, Changsha, 410011 Hunan, China

## Abstract

Abdominal aortic aneurysm (AAA) is defined as a progressive segmental dilation of the abdominal aorta and is associated with high mortality. The characterized features of AAA indicate several underlying mechanisms of AAA formation and progression, including reactive oxygen species production, inflammation, and atherosclerosis. Mitochondrial functions are critical for determining cell fate, and mitochondrial dynamics, especially selective mitochondrial autophagy, which is termed as mitophagy, has emerged as an important player in the pathogenesis of several cardiovascular diseases. The PARKIN/PARIS/PGC1*α* pathway is associated with AAA formation and has been proposed to play a role in mitochondrial dynamics mediated by the PINK/PARKIN pathway in the pathogenesis underlying AAA. This review is aimed at deepening our understanding of AAA formation and progression, which is vital for the development of potential medical therapies for AAA.

## 1. Background of AAA

Aortic aneurysm is universally characterized as the weakening of the aortic wall and leads to progressive dilatation [1]. Abdominal aortic aneurysm (AAA) most commonly affects the infrarenal part of the aorta; therefore, a widely used definition of AAA is a maximum infrarenal abdominal aortic diameter of ≥30 mm on ultrasonography or computed tomography (CT) imaging, although other definitions have been used in different studies, such as an infrarenal to suprarenal diameter ratio of 1.2 to 1.5 [1]. The weakened aortic wall of the AAA is pathologically typified with proteolytic destruction of extracellular matrix, inflammation, intense oxidative stress, and apoptosis of vascular smooth muscle cells (VSMCs) [2, 3]. Without intervention, the aneurysm progressively expands and leads to lethal aortic rupture [1, 4, 5]. Other less common complications include embolization, fistula formation, and iliac vein compression [1].

Aortic rupture commonly leads to bleeding into the retroperitoneum or abdomen with high mortality [5]. The maximum diameter of AAAs is the most significant predictive factor for the risk of aortic rupture [6, 7]; other factors predisposing to rupture include active smoking, rate of growth, aberrant biomechanical properties of the aneurysmal sac, and male sex [8]. Intraluminal hemodynamic conditions also influence AAA risk of rupture, and both peak wall stress and residual wall strength have been proposed as predictive parameters [9, 10]. The current recommendations for AAAs mostly refer to surgical treatment, including either open or minimally invasive surgery. These invasive interventions are indicated for large, asymptomatic AAAs and symptomatic or ruptured AAAs of any diameter [11, 12]. The critical diameter indicating surgery is 55 mm, as invasive surgery does not improve survival in patients with AAAs smaller than 55 mm in diameter [13, 14]. Small AAAs are only followed by periodic ultrasound surveillance until the diameter reaches 55 mm, when surgical repair is indicated. However, most patients present with small and asymptomatic AAA. Considering the high mortality of aortic rupture, it is of importance to identify aneurysms in early stages. The rapid advances of imaging techniques, including ultrasound and CT, have greatly improved the rate of detection [11]. Furthermore, defining patients with a high risk of developing AAAs is also helpful for early identification. According to the current understanding from observational studies, several risk factors have been identified, of which smoking is the most important modifiable risk factor. Cessation of smoking not only reduces the risk of developing AAA but also limits aneurysm expansion [15, 16]. Other risk factors include old age, male sex, family history of AAA, complications with other cardiovascular diseases, hypertension, and dyslipidemia [1, 5, 17, 18].

Even though significant advances have been made in the management of AAAs, the disease still poses a significant medical burden on early-stage management [19]. The benefit of invasive management is limited to patients with small, asymptomatic AAAs. However, an effective and specific medical therapy is not currently available [19, 20]. The lack of drug therapy that prevents aneurysm development or halts aneurysm expansion draws specific attention to improve our understanding of the underlying mechanism of AAA.

## 2. Current Theories of AAA Pathogenesis

### 2.1. Inflammation and Imbalance of ROS Production and Antioxidants

Inflammation plays an important role in both the development and the progression of AAA [5] and is not confined to only inflammatory AAA [21]. Chronic aortic inflammation may lead to the destruction of aortic tissue and VSMC dysfunction, and eventually apoptosis [5]. In agreement with the pathogenic theory of inflammation, in animal models, Kyoto Encyclopedia of Genes and Genomes (KEGG) network analysis demonstrated a significant upregulation of a wide range of immune processes, including cytokine–cytokine receptor interactions, leukocyte transendothelial migration, B cell and T cell signaling pathways, and natural killer cell-mediated cytotoxicity [22, 23]. Infiltration of innate and adaptive immune cells and their products is observed in the aortic wall [24, 25]. In addition, observational data from animal models or human specimens also support the notion that inflammation plays a causative role in the development and progression of AAAs. For example, apoptosis-associated speck-like protein containing a caspase recruitment domain (ASC) is highly expressed in adventitial macrophages. ASC deficiency reduced inflammatory cell infiltration and cytokine expression after vascular injury and attenuated the subsequent initial aneurysm formation [26]. Furthermore, the NLRP3 inflammasome may act as an essential mediator of vascular inflammation and subsequent AAA formation [26, 27].

The inflammation in AAA pathogenesis is associated with the production of reactive oxygen species (ROS) and oxidative stress [28, 29]. Mitochondrial oxidative stress from macrophages induces inflammation [28], which in turn enhances oxidative stress with resultant injury to tissues. Significant ROS production is one of the key features of the vascular wall in AAAs and is involved in the degeneration of the vascular wall. Higher levels of ROS such as O_2_^−^ and NOX, cyclo-oxygenase-2 (COX-2), and lipid peroxidation products are reported in biopsies of human aneurysmal aortas, and these products are responsible for exacerbating VSMC apoptosis and promoting proteolytic degradation of extracellular matrix (ECM) [30–32]. Dysregulation of the antioxidant protective mechanism has also been reported [33]. The imbalance of oxidants and antioxidants regulates ECM remodeling and promotes VSMC dysfunction, indicating a causative role in AAA development [33].

### 2.2. Atherosclerosis

Atherosclerosis is thought to play a role in the pathogenesis of AAA. Several risk factors are shared between AAA and atherosclerosis, such as smoking, family history, or complications with other atherosclerosis-related diseases. Ischemic heart disease and peripheral artery disease are also established risk factors for AAA prevalence and incidence. In atherosclerotic plaques, the loss of VSMCs in response to pathologic stimuli, such as oxygen-derived free radicals, leads to weakening of the arterial wall [5]. In addition, hemodynamic forces caused by plaques in AAAs induce phenotypic changes of VSMCs [5]. The nonproliferating differentiated VSMCs present with a contractile phenotype and allow normal vascular function [34], while VSMCs are stimulated to differentiate into the synthetic phenotype under circumstances of vascular injury, mechanical stress, and ROS stimulation. These synthetic VSMCs display a partial loss to matrix production and release matrix remodeling enzymes. Increased activity of matrix metalloproteinases-2 (MMP-2) from VSMCs and dysfunction of elastin and collagen lead to a weakening of matrix capacity and integrity [35, 36]. This abnormal function of VSMCs is a critical determinant in the pathogenesis of atherosclerosis [37] and promotes the formation of AAA.

### 2.3. Inherited Factors

In addition to the traditional consideration of environmental factors in the pathogenesis of aneurysms, a genetic role in AAA pathogenesis is supported by the increased risk of patients with a positive family history of AAA. A twin study suggested that additive genetic components may play a more significant role in AAA penetrance than environmental factors [38]. Several SNPs were reported to be associated to AAA directly. SNPs have been reported being individually associated with risk of AAA [5, 39]. For example, the SNP of rs6511720 in LDL receptor and SNP of rs602633 in Sortilin 1 were reported to be associated with lower AAA risk [39]. These risk alleles are associated with matrix remodeling, immune function, and lipid metabolism [39].

## 3. A Potential Role of Mitochondrial Function in the Pathogenesis of AAA

Based on our current understanding of the underlying mechanism of AAAs, several pharmacological therapies have been proposed for preventing or halting the development or growth of AAA. These include widely used preventive medicines, such as statins, antiplatelet drugs, or corticoids, targeting the potential pathogenic pathways in a general manner. Other attempts include more specific targets for critical signaling pathways (including AKT signaling pathway and Notch signaling pathway), kinases (such as c-Jun N terminal kinase (c-JNK) and extracellular signal-related kinase), or cytokines (such as interleukin-1*β*) [19, 20]. However, a large number of drugs that were expected to be effective in preclinical animal experiments failed in clinical trials eventually. One of the most heated drugs is doxycycline, which strongly inhibited AAA formation in AngII-induced AAA models through reducing matrix metalloproteinase (MMP) activity [40], but it failed in large randomized, placebo-controlled, double-blind trial, as patients did not gain benefit during AAA progression [41]. The current application of genome analysis may provide several new insights into the area. According to the gene expression profiling analysis of differentially expressed genes (DEGs) between AAA samples and normal controls, a total of 436 DEGs were identified using Gene Ontology (GO) and KEGG analyses [42], and four different clusters were identified from the protein–protein interaction network [42]. In addition to the known and traditional pathways involved in the response to viral infection and the defense response indicative of the role of inflammation during AAA, one of the four clusters was associated with mitochondria-associated functions and the oxidative phosphorylation subpathway [42]; a potential role of mitochondrial function in the pathogenesis of AAA was thus proposed.

### 3.1. Mitochondrial Dynamics

The maintenance of mitochondrial function is crucial for normal cell physiology not only in the aspect of ATP production but also in the role of regulating cell death and survival through integrating cellular signals. The mitochondrion is also the primary source of ROS, triggering oxidative stress and the downstream changes of cell fate [43].

Several homeostasis mechanisms promote proper mitochondrial functions in normal cells. Mitochondrial dynamics, which includes mitochondrial fission, fusion, biogenesis, and mitophagy, determines the morphology, quality, and abundance of mitochondria [43].

Mitochondrial fusion involves changes during mitochondrial morphologic changes and is responsible for the exchange of mitochondrial matrix and DNA between individual mitochondria [44]. Mitochondrial fusion is a multistep process including the sequential fusion events of the outer mitochondrial membrane (OMM) and inner mitochondrial membrane (IMM). OMM fusion is mediated by mitofusin 1 and 2 (MFN1/2), while the fusion of IMM is regulated by the GTPase Optic Atrophy 1 (OPA1) [43, 45]. Fission is essential for maintenance and repair as it facilitates the removal of damaged components. The recruitment of the GTPase Dynamin-related protein 1 (DRP1) is crucial for mitochondrial fission and is mediated by mitochondrial fission 1 protein (FIS1), mitochondrial fission factor (MFF), and mitochondrial dynamic proteins of 49 and 51 kDa (MiD49/51) [43, 45]. DRP1 is dephosphorylated and recruited on the outer mitochondrial surface, and this process initiated mitochondrial fission. Subsequently, DRP1 oligomerizes and induces GTP hydrolysis-mediated membrane constriction [46]. Mitochondrial biogenesis is responsible for incorporating new and healthy units into the mitochondrial network and for increasing mitochondrial mass [47]. Peroxisome proliferator-activated receptor coactivator 1 *α* (PGC1*α*) [48] and its downstream nuclear respiratory factors 1 and 2 (NRF1 and 2), mitochondrial transcription factor A (TFAM), and voltage-dependent anion channel (VDAC) are critical in modulating mitochondrial biogenesis [49]. Among these factors, PGC1*α* is a nuclear encoding factor that initiates mtDNA transcription. Mitophagy is essential for eliminating the aged or damaged mitochondria in response to changes in the cellular environment [50]. The canonical regulatory pathway of mitophagy to date involves the PINK1/PARKIN pathway. The serine/threonine kinase, phosphatase, and tensin homologue- (PTEN-) induced kinase 1 (PINK1) accumulate on the OMM in response to a trigger that initiates selective mitophagy, such as ROS [51], Mitochondrial Permeability Transition Pore (mPTP) opening [52], and loss of mitochondrial membrane potential [53]. The accumulation of PINK1 recruits PARKIN, an E3 ubiquitin ligase which ubiquitinates several downstream substrates and leads to the ensuing autophagy processes [54]. Another noncanonical pathway involves receptors that mediate mitophagy, such as BCL-2- (B cell lymphoma protein-2-) related proteins BNIP3 (BCL2 Interacting Protein 3), FUN14 domain-containing protein 1 (FUNDC1), and the OMM protein Bcl2-like protein 13 (Bcl2-L-13). These receptors interact with LC3 (Light Chain 3) without the need for another adaptor protein [51].

**Figure 1:**
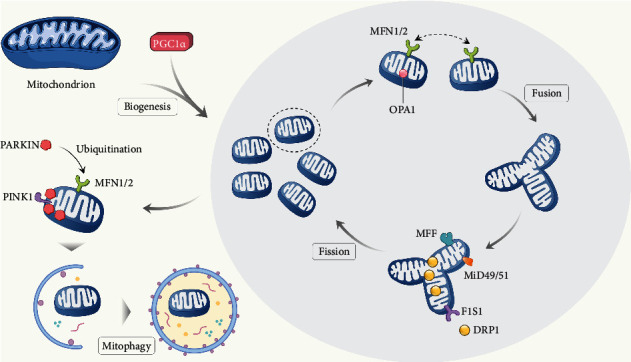
Mitochondrial dynamics. Mitochondrial dynamics includes the process of mitochondrial biogenesis, fusion, fission, and mitophagy and determine the proper abundance and function of mitochondria. (1) PGC-1*α* regulates mitobiogenesis and initiates mtDNA transcription through its downstream factors, such as NRF1/2. (2) Mitofusion 1/2(MFN1/2) on the outer mitochondrial membrane and optic atrophy 1 (OPA1) on the inner mitochondrial membrane regulates mitochondrial fusion. (3) DRP1 serves to constrict mitochondrion physically and uses FIS1 as mitochondrial targets to form the fission complex, and MFF and MiD49/51 also participate in mitochondrial fission. (4) Canonical regulation of mitophagy includes the PINK1/PARKIN pathway; PINK1 recruits PARKIN on mitochondrion where PARKIN ubiquitinates downstream proteins and initiates the form of mitophagy.

The balance between fusion and fission is sustained to maintain the proper function of mitochondria, while biogenesis and mitophagy are essential for mitochondria renewal and elimination of dysfunctional mitochondria (Figure 1).

## 4. Mitochondrial Function in AAA

Mitochondrial dynamics is critical for mitochondrial health and quality control. Dysfunction of mitochondria participates in the pathogenesis of several cardiovascular diseases [43], such as diabetic cardiomyopathy [55], myocardial infarction, and ischaemia/reperfusion [56]. Mitochondrial dysfunction, including disturbed mitochondrial dynamics including mitochondrial fusion, fission, mitobiogenesis, and mitophagy, is thought to contribute to the pathogenesis underlying the formation and development of AAA.

### 4.1. Mitochondrial Fusion and Fission and AAA

Mitochondrial fusion and fission are tightly regulated and together determine the shape and functions of mitochondria. Subcutaneous infusion of angiotensin II is one of the main methods used to induce AAA in mice, which recapitulates several important features of human AAA, including inflammation and promotion by significant risk factors of human AAA, such as male sex and smoking. Exposure to angiotensin II in cultured rat aortic VSMCs induces mitochondrial fission [57], which can be prevented by the putative DRP1 inhibitor Mdivi-1 (mitochondrial division inhibitor 1) [57]. A recent study has found that DRP1 expression was enhanced in human AAA samples compared to age-matched healthy controls [58, 59]. Furthermore, DRP1 inhibition by Mdivi-1 protects apolipoprotein E-deficient mice infused with angiotensin II from AAA development, which was assessed by the measurement of external and internal diameters of the abdominal aorta as well as by histological observation [58]. Also, the heterozygous expression of DRP1 presents a protective role in AAA development. DRP1-mediated fission leads to a decrease in the number of mitochondria in AAA tissues, which is attenuated by Mdivi-1. Mdivi-1 also attenuates the inflammatory phenotype in abdominal aortic VSMCs [58]. The protection of AAA by DRP1 inhibition is associated with a reduced stress response and senescence. Senescent phenotype was seen in both mouse AAA models and human AAA samples, and its attenuation by Mdivi-1 was confirmed in mouse AAA models treated with AngII and *β*-aminopropionitrile [58]. Therefore, DRP1-mediated mitochondrial fission potentially promotes proinflammatory phenotypic changes of VSMCs and contributes to the pathogenesis of AAA development.

### 4.2. Mitochondrial Biogenesis and AAA

Mitochondrial biogenesis is the process that increases mitochondrial mass and is involved in the control of cell metabolism and signal transduction. Recent studies highlight that PGC1*α*, the main regulator of mitochondrial biogenesis and protector from oxidative stress [59], also regulates VSMC migration and matrix formation. Oxidative stress and VSMC dysfunction contribute to the pathogenesis of AAA, and peroxisome proliferator-activated receptor-*γ* (PPAR*γ*) is involved in AAA formation; PPAR*γ* is a member of the nuclear receptor superfamily of ligand-dependent transcription factors, which increases gene expression when binding to DNA; loss of PPAR*γ* expression has been demonstrated to promote AAA, and activation of PPAR*γ* attenuates AAA formation [49, 60, 61]. Therefore, the role of mitochondrial biogenesis in AAA formation is emerging. PGC1*α* promotes mitochondrial biogenesis and acts as a coactivator of nuclear respiratory factor-1 (NRF-1) or/and nuclear respiratory factor-2 (NRF-2), therefore increasing the expression of mitochondrial genes involved in oxidative phosphorylation such as human cytochrome c [62]. In human AAA specimens, the gene expression of *PPARGC1A* was reduced by 51%, and the gene expression levels of VDAC and TFAM were reduced significantly concomitant with the *PPARGC1A* expression compared to the healthy control [49]. The ratios of the expression of Cytochrome B and Cytochrome C oxidase vs. that of *β*-actin were also reduced in another study. The ratios act as a marker for mitochondrial biogenesis, and this indicates that mitochondrial biogenesis is disturbed in AAA [63]. In support of the role of PGC1*α*-mediated mitochondrial biogenesis in aneurysm formation, Pluijm et al. generated a Fibulin-4^R/R^ mouse model as a progressive ascending aneurysm formation model [64]. Fibulin-4 is a secreted glycoprotein that is critical for structural integrity and elasticity of the aortic wall, and haploinsufficiency of Fibulin-4 compromises the integrity of aortic wall leading to aneurysm formation [65]. The mRNA levels of PGC1*α* were significantly downregulated in Fibulin-4^R/R^ aortas, and the activity of PGC1*α* was also lower in Fibulin-4^R/R^ VSMCs which is confirmed in luciferase-based assay. Furthermore, the activation of PGC1*α* after Forskolin treatment significantly increased the proliferation rate of Fibulin4^R/R^ VSMCs and rescued the decrease in OCR [64]. Taken together, disturbance of mitochondrial biogenesis participates in the pathogenesis of AAA, and dysfunction of mitochondrial respiration is linked to PGC1*α* regulation in AAA [49, 64].

### 4.3. Mitophagy and AAA

There are limited data with respect to the direct role of mitophagy in AAA pathogenesis, though the role of mitophagy in cardiovascular pathology is emerging [46]. The ability of mitophagy to remove damaged and dysfunctional mitochondria may protect cardiomyocytes from prolonged cardiac stress. Indeed, it has been demonstrated that mitophagy is necessary for cardiomyocyte adaptation to changes in cardiac load [46]. In addition, impaired PINK1/PARKIN-mediated mitophagy directly leads to myocardial dysfunction [43]. *PINK1^−/−^* mice develop early left ventricular dysfunction and pathological cardiac hypertrophy [66], which is associated with increased oxidative stress, and PINK1 protein levels are remarkably reduced in patients with advanced heart failure [66]. Based on current understanding of mitophagy, it may participate in the pathogenesis of AAA in several ways.

Mitophagy is a crucial process that helps eliminate dysfunctional mitochondria before they cause damage or trigger cell death, thereby maintaining mitochondrial homeostasis, and may reduce oxidative damage or ROS production [54]. The disturbed function of mitophagy obviously leads to the accumulation of dysfunctional mitochondria and insufficient ATP production and results in cell death eventually, provoking excessive ROS production. Mitochondrial ROS (mtROS) production activates redox-sensitive transcription factors and facilitates the production of proinflammatory factors such as interleukin-6 (IL-6), which is abundant in AAA tissues and increases in circulating level [20, 67, 68]. Mitochondrial ROS also recruit ASC and caspase 1 precursor, promoting the maturation of IL-1*β* and IL-18 [69]. The NLRP3 inflammasome has been reported as an initiating mediator for AAA formation [27, 28]. Inhibition of mitophagy facilitates the activation of the NLRP3 inflammasome [70]. Oxidized mtDNA released into cytosol binds and activates the NLPR3 inflammasome directly in macrophage [67, 71]; activation of macrophage inflammasome is involved in hyperhomocysteinemia-aggravated AAA formation, as observed in vitro and in AngII-infused apolipoprotein E-deficient mice [2].

Dysfunctional mitophagy also plays a significant role in atherosclerotic plaque destabilization and overall plaque development [72] and seems to depend on certain cell types involved in the pathogenesis of atherosclerosis. VSMCs isolated from carotid plaques showed increased mitophagy levels, accompanied by elevated expression of PINK1, compared to healthy VSMCs [73]. Defective mitophagy in VSMCs leads to cell senescence, presenting a senescence-related secretory phenotype characterized with elevated ability of migration and loss of contractile proteins such as *α*-smooth muscle actin and calponin [72, 74, 75].

Impaired mitophagy in macrophages induces polarization into a proinflammatory phenotype (termed as M1) of macrophages [76] and accelerates atherosclerotic plaque development. Plaques from these models develop an unstable phenotype characterized with an increase in the necrotic core area and apoptosis [72].

The dysfunction of VSMCs is a significant hallmark in AAA, as dysfunction or apoptosis of VSMCs leads to the release of enzymes that are responsible for the degradation of ECM [2, 3]. During pathological stimuli, such as cholesterol, VSMCs acquire proliferative and migratory capacity, accompanied by a loss of SMC markers such as actin, and expression of macrophage antigens like CD68 and Arginase 1 [35]. Platelet-derived growth factor (PDGF) promotes the dedifferentiation of VSMCs and induces the synthetic phenotype. PDGF also induces mitophagy in a time-dependent manner as the formation of LC-II increases [35, 72, 77]. There is a definite link between mitochondrial dysfunction and dedifferentiation of VSMCs. Deficiency in mitochondrial protein polymerase interacting protein 2 (Poldip2) induces metabolic reprogramming with repressed mitochondrial respiration and increased glycolytic activity [35] and leads to significant upregulation of proteins from the contractile apparatus, which means that VSMCs present with a highly differentiated phenotype [35]. Apelin is a family of novel adipokine that activates receptors present on VSMCs and cardiomyocytes and elicits cardiovascular effects on experimental animals [78, 79]. Apelin-13 is one of the endogenous isoforms in human cardiac tissue that mediates vasodilatation, vasoconstriction, and cardiac contractility [79]. Apelin-13 promotes human aortic VSMC proliferation [34], and this promotion is related to increased mitophagy. In response to the Apelin-13 induction, the expression levels of PINK1, PARKIN, and VDAC1 are effectively enhanced; blocking Apelin-13 reversed this stimulatory effect, and the downregulation of PINK1/PARKIN ameliorates the enhanced proliferative capacity in response to Apelin-13 in vivo demonstrated in PINK1^−/−^ mice [34].

We speculate that the PINK1/PARKIN pathway plays a role in the mitochondrial dynamics in AAA. It is well established that mitophagy induces a metabolic shift and phenotypic transition in VSMCs and regulates mitochondrial ROS production, inflammation, and atherosclerosis development. It is reasonable to propose that disturbed mitophagy contributes to the pathogenesis of aneurysm. Also, we emphasize the central role of the PINK1/PARKIN pathway, as the E3 ubiquitin ligase PARKIN ubiquitinates several downstream substrates not confined to a role in mitophagy. MFNs are also ubiquitinated by PARKIN for proteasomal degradation [43]. Mitochondrial fusion and fission are tightly mediated by dynamin-like GTPases (DRP1 and MFN1/2, respectively). These GTPases all possess a cytosolic domain that is targeted for ubiquitin [80]. It is clear that the ubiquitin/proteasome system is crucial for mitochondrial quality control and the regulation of mitochondrial morphology [81]. The expression and recruitment of DRP1 lead to mitochondrial fission in a PINK1-dependent manner and to mitophagy [82, 83]. This ubiquitylation process leads to the prevention of fusion and the promotion of fission and mitophagy [46]. In addition, it has been demonstrated that PGC1*α* is regulated by PARKIN-interacting substrate (PARIS) [84]. PARIS is a protein that contains a Krüppel-associated box (KRAB) at its N-terminus and a C2HC/C2H2 type zinc finger at its C-terminus and acts as a major transcriptional repressor of PGC1*α* [84]. PARIS interacts with PARKIN directly and is ubiquitinated by PARKIN for degradation under normal conditions. Accumulation of PARIS due to PINK1/PARKIN dysfunction or overexpression of PARIS leads to significant decreases in the mitochondrial size, number, and protein expression [85, 86].

## 5. Conclusion

Mitochondrial dynamics is a process that is crucial for mitochondrial homeostasis [51]. The role of dysfunctional mitochondria or disturbed mitochondrial dynamics in cardiovascular diseases in several pathologic conditions, such as cardiomyopathy and cardiac ischemia/reperfusion, is emerging [54]. AAA is one of the most puzzling vascular diseases to date, as there is no medication available despite advances in surgical intervention having saved a large number of lives [20]. There is an urgent need to deepen our understanding of the pathogenesis of AAA formation and development, to develop new medications for the treatment and/or prevention of AAA. Given its significance in mitochondrial quality control, we propose that disturbed mitochondrial dynamics contributes to the pathogenesis of AAA and underlines the central role of the PINK1/PARKIN pathway during the pathogenesis of aneurysms based on currently available evidence. Direct evidence implicating mitophagy in AAA formation, as well as potential pharmacological interventions derived from mitochondrial dynamics, is being anticipated.

## Data Availability

There is no underlying data as declared.
